# La nécrobiose lipoïdique: à propos d’un cas: Necrobiosis lipoidica: about a case

**DOI:** 10.11604/pamj.2018.29.5.14226

**Published:** 2018-01-03

**Authors:** Nadia El Haddad, Hanan El Ouahabi

**Affiliations:** 1 Hôpital Ibn Al khatib, Fès, Maroc; 2 Centre Hospitalier Universitaire Hassan II, Fès, Maroc

**Keywords:** Nécrobiose lipoidique, diabète type 1, dermatose granulomateuse, Necrobiosis lipoidica, type 1 diabetes, granulomatous dermatosis

## Abstract

Necrobiosis lipoidica is a rare granulomatous dermatosis. It occurs in 0.3-1.2% of diabetic patients mainly at the level of the leg. The lesions appear as erythematous plaques with central depressions. We here report the case of a 21-year old female patient with a 6-year history of type 1 diabetes presenting with a 1-year history of well circumscribed, symmetrical and asymptomatic erythematous plaques with atrophic yellowish center at the level of the anterior compartments of the legs (A, B). The diagnosis of necrobiosis lipoidica was suspected, a biopsy was performed which confirmed the diagnosis. The patient had a granulomatous inflammatory reaction with palisade-like border disposed around poorly delimited foci of altered connective tissue on the entire dermis layer. The inflammatory reaction was also characterized by multinucleate giant cells, dendrocytes and lipid deposits. Local corticosteroid therapy was prescribed. Patient's evolution was marked by the persistence of the lesions.

## Image en médecine

La nécrobiose lipoïdique est une dermatose granulomateuse rare. Elle est rapportée chez 0,3 à 1,2% des diabétiques, localisées préférentiellement au niveau de la jambe, les lésions apparaissent comme des plaques érythémateuses, avec des dépressions centrales. Nous rapportons l'observation d'une patiente âgée de 21 ans connue diabétique type 1 depuis 6 ans et qui présente depuis 1 an des plaques érythémateuses bien circonscrites avec un centre jaunâtre atrophique au niveau des faces antérieures des jambes symétriques et asymptomatique (A, B). Le diagnostic de nécrobiose lipoïdique a été évoqué, une biopsie a été réalisée et l'a confirmé. Elle a montré une réaction inflammatoire granulomateuse sur toute l'épaisseur du derme à bordure palissadique disposée autour de foyers mal limités de stroma conjonctif altéré. Des cellules géantes plurinucléées et des dendrocytes étaient présents, des dépôts lipidiques étaient reconnus. Une corticothérapie locale a été prescrite chez notre patiente. L'évolution a été marquée par une persistance des lésions.

**Figure 1:**
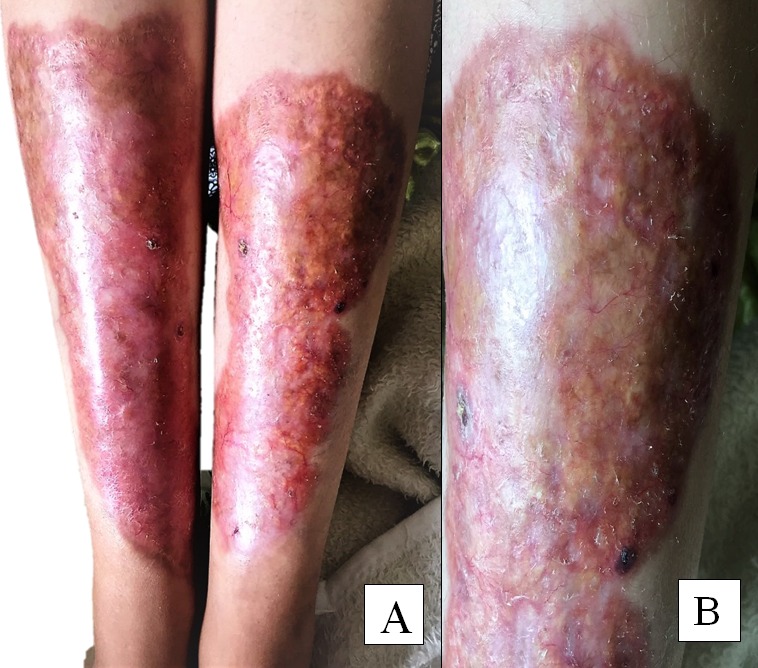
(A,B) plaques érythémateuses bien circonscrites des deux jambes avec un centre jaunâtre (dépôts lipidiques)

